# Impact of Social Media Addiction Among Medical Students on Their Social Interaction, Well-Being, and Personality: A Comparative Study

**DOI:** 10.7759/cureus.70526

**Published:** 2024-09-30

**Authors:** Amarbir Singh, Suprakash Chaudhury, Bhushan Chaudhari

**Affiliations:** 1 Department of Psychiatry, Dr. D. Y. Patil Medical College, Hospital and Research Center, Dr. D. Y. Patil Vidyapeeth (Deemed to Be University), Pune, IND

**Keywords:** loneliness, medical college, medical students, personality, social anxiety, social interaction, social media addiction, well-being

## Abstract

Background

Social media addiction has emerged as a growing concern, particularly among young adults, including medical students who face unique stressors and demands. The widespread usage of social media platforms can lead to addictive behaviors affecting mental health, academic performance, and interpersonal relationships. Understanding the relationship between social media addiction, personality traits, social interaction, and overall well-being is crucial for developing effective interventions to support this vulnerable group.

Aim

To study the relationship of social media addiction with social interaction, well-being, and personality in medical students.

Materials and methods

A cross-sectional study was conducted with 300 students from a medical institution, including both undergraduate and postgraduate levels. Data were collected through an online survey, administered via Google Forms. Social Media Addiction Scale-Student Form (SMA-SF), Social Interaction Anxiety Scale and Social Phobia Scale, Medical Student Well-Being Index (MSWBI), Three-Item Loneliness Scale (T-ILS), and Short Big Five Inventory Scale (BFI-10) were applied to all the participants.

Results

A total of 292 medical students participated in the study, comprising 122 males and 170 females. The analysis revealed that both male and female students exhibit similar levels of susceptibility to social media addiction, as evidenced by comparable scores on measures of social media addiction, social interaction anxiety, social phobia, loneliness, well-being, and personality traits. Regression analysis identified time spent on social media and agreeableness as significant predictors of social media addiction, with no signs of multicollinearity. These findings indicate that male and female medical students share similar psychological profiles, highlighting critical factors that influence social media usage within this population.

Conclusion

This study highlights a significant prevalence of social media addiction among medical students, affecting 76.7% (n = 224) of participants, with slightly higher rates observed among females (n = 132, 78%) compared to males (n = 92, 72%). This addiction correlates with adverse psychological traits such as heightened social interaction anxiety, social phobia, and loneliness, along with personality traits like neuroticism. Both genders exhibit similar susceptibility to addiction, influenced by factors such as time spent socializing and agreeableness. Addressing these findings through targeted interventions could improve medical students' overall well-being and mental health outcomes, underscoring the need for further research and effective strategies in academic settings.

## Introduction

In today's digital age, social media is pivotal in shaping how people interact and present themselves. Research indicates that social media can have both beneficial and detrimental effects. On the positive side, platforms enable individuals to connect across distances, build social networks, and engage in self-expression, fostering inclusivity and social engagement [1]. However, there are potential negative consequences, such as increased social comparison, pressure to present an idealized version of oneself, and adverse impacts on self-esteem and mental health [2]. Thus, while social media offers valuable tools for communication and identity formation, usage patterns heavily influence its effects.

Medical students who navigate a particularly demanding academic journey are not exempt from these influences. Although social media can provide significant benefits in terms of connectivity, information sharing, and collaboration, there is growing concern about the potential for addiction and its associated consequences. As this group faces unique stressors and challenges, understanding the impact of social media on their well-being is crucial.

Social media addiction refers to the compulsive and uncontrollable use of social media, leading to preoccupation with online activities and impairments in other life areas [3]. It has also been associated with negative outcomes such as decreased academic performance, impaired relationships, and mental health issues, including depression, anxiety, and low self-esteem [4]. Medical students facing rigorous curriculums and high stress may use social media as a coping mechanism, potentially impacting their well-being, social interactions, and personality traits [5].

Excessive social media usage can negatively affect well-being by promoting sedentary behavior, disrupting sleep, and increasing feelings of envy and loneliness [6]. The impact on social interactions includes reduced face-to-face communication and meaningful relationships [7]. Personality traits such as extraversion, agreeableness, neuroticism, openness to experience, and conscientiousness are also explored in social media addiction [8], but the specific impact on medical students is yet underresearched.

This study investigates the relationships between social media addiction and its effects on social interaction, well-being, and personality among medical students. Understanding these connections is crucial for addressing the challenges faced by future healthcare providers in maintaining their personal and professional growth.

## Materials and methods

Study design

This cross-sectional analytical study was carried out at Dr. D. Y. Patil Medical College, Hospital and Research Center located in a suburban area of Pune, India. The study proposal was submitted, and ethical clearance was obtained from the Institutional Ethics Sub-Committee of Dr. D. Y. Patil Medical College, Hospital and Research Center (approval no. IECS/PGS/2022/55) before the study began. This was documented in letter no. I.E.S.C./P.G. S/2022/55, dated September 28, 2022.

Sample size

According to an earlier study, the prevalence of social media addiction is 17.7% [9]. To determine the sample size for the study, Fisher's formula was used: \[ n = \frac{z^2 \cdot p \cdot (1 - p)}{d^2}\]​ where n represents the sample size, z is the z-score corresponding to a 95% confidence level (1.96), p is the expected prevalence or proportion, and d is the margin of error (0.05). Substituting these values into the formula, we have: \[n = \frac{1.96 \times 1.96 \times 0.177 \times 0.823}{0.025^2}\] This calculation results in a sample size of approximately 224.

Sample

Participants for the study were selected through convenience sampling from undergraduate and postgraduate medical students at a tertiary care hospital based on specific inclusion and exclusion criteria. The study will include male and female medical students willing to provide informed consent. Written informed consent was secured from all participants recruited after explaining the purpose and design of the study. However, students with a history of psychiatric disorders and those who are not willing to give consent will be excluded from participation. This approach ensures a diverse yet ethically sound participant pool, allowing for a thorough and reliable analysis of the research topic.

Collection of data

Participants for the study were chosen from medical students at a tertiary care hospital based on specific criteria for inclusion and exclusion. We distributed online survey forms to these students and collected their responses. The survey included questions on socio-demographic details such as age, sex, relationship status, age at first phone, and time spent on social media. Other questionnaires included SMS-SF, Social Interaction Anxiety Scale-6 (SIAS-6), Social Phobia Scale-6 (SPS-6), Three-Item Loneliness Scale (T-ILS), Medical Student Well-Being Index Scale (MSWBI), and BFI-10.

Method

Medical students who met the study's inclusion criteria were invited to participate and informed written consent was obtained from each participant. An online survey, such as Google Forms, was distributed to 300 students at a medical college, and 292 responses were received. The aims and objectives of the study were clearly explained to all participants, who were subsequently interviewed, with clinical data being recorded. To assess social media addiction, various scales were administered to those who agreed to participate in the study.

Tools

The study utilized several assessment tools to gather data on the participants. First, a sociodemographic and clinical data sheet was used, which was a self-designed proforma that included questions about the demographic characteristics of the subjects. This provided a baseline understanding of the participants' backgrounds.

To assess social media addiction, the Social Media Addiction Scale Student Form (SMAS-SF) was employed. This scale is composed of 29 items, organized into four sub-dimensions, and rated on a five-point Likert scale. The first five items assess virtual tolerance, items 6 to 14 evaluate virtual communication, items 15 to 23 focus on virtual problems, and items 24 to 29 measure virtual information. The scale's scores range from a minimum of 29.8 to a maximum of 145. With an internal consistency coefficient of 0.93, this scale is considered reliable and valid [10].

To evaluate social anxiety, the study utilized the SIAS and the SPS. These scales examine two distinct kinds of social anxiety: anxiety during dyadic interactions and fear of being seen in performance circumstances. Rather than the original 20-item scales, we used the shortened six-item version scale (SIAS-6 and SPS-6) created by Peters et al. Each question on these shorter measures is scored on a five-point Likert scale, with options ranging from zero (not at all) to four (very much). The Korean versions of the SIAS-6 and SPS-6 have been validated; an SIAS-6 total score of 12 or above and an SPS-6 total score of nine or above indicate social interaction anxiety and social phobia, respectively [11,12].

The MSWBI was also included as a screening instrument for detecting psychological distress in medical students and identifying individuals at risk for severe outcomes who may need personalized treatment. Cronbach's alpha is 0.69, indicating adequate internal consistency. The Pearson correlation coefficients and receiver operating characteristic (ROC) curves revealed that each item on the MSWBI had sufficient convergent and discriminant validity [13,14].

Loneliness was assessed using the T-ILS, which was established by Hughes et al. and is designed to assess levels of loneliness. Unlike the original UCLA Loneliness Scale, the T-ILS employs second-person wording in its items. Each item is assessed on a scale from one to three, producing scores between three and nine points. Higher scores on this scale signify a more pronounced sense of loneliness. The original T-ILS has good internal consistency and a robust association (r = 0.82) with the original UCLA Loneliness Scale, making it especially useful for epidemiological investigations [15].

Finally, to measure the big five personality traits - extraversion, agreeableness, openness, conscientiousness, and neuroticism - the study used the BFI-10, as employed by the World Values Survey and developed by Rammstedt and John. The BFI-10 has ten items, with each personality feature represented by two positive and negative sentences. Participants evaluated each statement using a five-point Likert scale, where one represents "strongly disagree" and five means "strongly agree" [16].

Statistical analysis

The data was entered into an Excel spreadsheet, and the recorded scores were compiled. Appropriate statistical analyses, including descriptive and inferential statistics, were conducted using IBM SPSS Statistics for Windows, Version 23 (Released 2015; IBM Corp., Armonk, New York, United States). Descriptive statistics were presented as means, standard deviations, percentages, and other relevant measures. The tests of significance used included the chi-square test, t-test, Spearman’s rank correlation, and Mann-Whitney U test, with a p-value of less than 0.05 considered significant.

## Results

A total of 292 students completed the questionnaires, with 122 male and 170 female participants.

Socio-demographic details showed that the two groups were not significantly different in age, relationship status, time spent on social media, age at first phone, and use of phone before sleep. This suggests that these socio-demographic factors are comparable across the groups and unlikely to account for any observed differences in the study outcomes, as given in Table 1.

**Table 1:** Comparisons of socio-demographic details of male and female medical students The chi-square test is used to analyze the association between males and females. p < 0.05 is significant

Characteristics		Males	Females	Chi sq.	p-value
Age distribution	≤23 years	101 (82.8%)	150 (88.2%)	1.7469	0.1862
	≥24 years	21 (17.2%)	20 (11.8%)		
Relationship status	Single	102 (83.6%)	132 (77.6%)	1.6195	0.4449
	Committed	17 (13.9%)	33 (19.4%)		
	Married	3 (2.5%)	5 (2.9%)		
Time spent on social media	<2 hours	44 (36.1%)	52 (30.6%)	4.0875	0.1295
	2-4 hours	59 (48.4%)	75 (44.1%)		
	>4 hours	19 (15.6%)	43 (25.3%)		
	Mean	1.7951	1.8836		
	SD	0.69161	0.72757		
Age at first phone	<10 years	4 (2.4%)	3 (1.8%)	1.5845	0.1295
	10-15 years	44 (25.9%)	60 (35.3%)		
	16-20 years	72 (42.4%)	106 (62.4%)		
	>20 years	2 (2.2%)	1 (0.6%)		
	Mean	15.8443	15.9247		
	SD	3.26013	2.71330		
Use of phone before sleep	Yes	108 (88.5%)	14 (11.5%)	0.6405	0.4235
	No	145 (85.3%)	25 (14.7%)		

The data analysis indicates no significant differences between males and females across various measures, including social media addiction, social interaction anxiety, social phobia, time spent on social media, mental well-being, agreeableness, conscientiousness, neuroticism, and openness to experience. However, a significant difference is observed in extraversion (p = 0.009), with males scoring higher than females. Additionally, there is a trend toward significance in mental well-being and agreeableness, with females showing slightly higher scores than males, as given in Table 2.

**Table 2:** Comparisons of scores obtained by students on the Social Media Addiction Scale-Student form, Social Interaction Anxiety Scale and Social Phobia Scale, Three-Item Loneliness Scale, Medical Student Well-Being Index Scale, and Short Big Five Inventory Scale SMA-SF: Social Media Addiction Scale-Student Form; SIAS-6: Social Interaction Anxiety Scale-6; SPS-6: Social Phobia Scale-6; T-ILS: Three-Item Loneliness Scale; MSWBI: Medical Student Well-Being Index Scale Mann-Whitney U test is used to compare the scores of males and females. p < 0.05 is significant

Scales	Gender	Mean	Median	SD	Mann-Whitney U	p-value
SMA-SF	Males	73.8115	80.5000	21.91167	10191.000	0.801
	Females	73.8356	79.0000	22.15892		
SIAS-6	Males	6.9180	6.0000	5.69702	9591.000	0.270
	Females	7.3116	7.0000	5.74685		
SPS-6	Males	7.0164	6.0000	5.95851	10357.500	0.986
	Females	7.0445	6.0000	6.01956		
T-ILS	Males	5.2623	6.0000	1.71914	9953.500	0.543
	Females	5.3767	6.0000	1.88552		
MSWBI	Males	3.0328	3.0000	2.40156	9053.00	0.062
	Females	3.3493	4.0000	2.42295		
Extraversion	Males	6.5000	6.0000	1.58114	8606.000	0.009
	Females	6.2123	6.0000	1.65269		
Agreeableness	Males	6.5328	6.0000	1.43270	9166.000	0.073
	Females	6.7021	6.0000	1.44674		
Conscientiousness	Males	6.5738	6.0000	1.33558	9975.00	0.544
	Females	6.5240	6.0000	1.29343		
Neuroticism	Males	5.6230	6.0000	1.41613	9438.00	0.163
	Females	5.8219	6.0000	1.51140		
Openness	Males	6.2623	6.0000	1.24518	9778.500	0.364
	Females	6.3356	6.0000	1.30174		

The table on social media addiction by gender reveals varying levels among males and females. While no statistically significant difference appears in the prevalence of no addiction (23% males vs. 22% females, p = 0.170092), females show slightly higher rates of mild addiction (64% vs. 55% males). Moderate addiction is marginally higher in males (16% vs. 10% of females), while severe addiction is notably higher in females (4% vs. 1% of males). These trends suggest potentially higher addiction levels among females, especially in milder and severe categories; further statistical analysis is needed to confirm these differences conclusively, as given in Table 3.

**Table 3:** Social Media Addiction Scale-Student form scoring gender wise The chi-square test is used to analyze the association between males and females. p < 0.05 is significant

Addiction	Male	Female	Total	Chi sq.	p-value
No (29-58)	30 (23%)	38 (22%)	68 (23.3%)	5.0234	0.170092
Mild (59-88)	71 (55%)	109 (64%)	180 (61.6%)		
Moderate (89-118)	20 (16%)	17 (10%)	37 (12.7%)		
Severe (>118)	1 (1%)	6 (4%)	7 (2.4%)		

The study indicates a weak positive correlation (Spearman’s Rho = 0.149, p = 0.11) between social media addiction and time spent socializing among participants. This suggests that higher levels of social media addiction may slightly correlate with increased time spent socializing, though the relationship is not statistically significant at the conventional level.

The SIAS and SPS were analyzed for their correlations with various psychological and behavioral measures. Both scales showed significant positive correlations with loneliness and neuroticism (NEURO), indicating that higher levels of social anxiety and phobia are associated with increased feelings of loneliness and higher neurotic tendencies. Additionally, both SIAS and SPS were negatively correlated with well-being (MSWBI), extraversion (EXTRA), agreeableness (AGREE), and conscientiousness (CONSC), suggesting that greater social anxiety and phobia are linked to lower levels of well-being and personality traits such as extraversion, agreeableness, and conscientiousness as given in Table 4.

**Table 4:** Correlations of social media addiction with Social Interaction Anxiety Scale and Social Phobia Scale T-ILS: Three-Item Loneliness Scale; MSWBI: Medical Student Well-Being Index Scale; NEURO: Neuroticism; EXTRA: Extraversion; AGREE: Agreeableness; CONSC: Conscientiousness Spearman's Rho test is used for correlation between various factors. p < 0.05 is significant

Factor	Spearman’s Rho (SIAS)	p-value (SIAS)	Spearman’s Rho (SPS)	p-value (SPS)
Social phobia	0.813	0.0001	-	-
Social Interaction Anxiety	-	-	0.813	0.0001
Loneliness (T-ILS)	0.498	0.0001	0.439	0.0001
Well-being (MSWBI)	0.182	0.002	0.232	0.0001
Neuroticism (NEURO)	0.261	0.0001	0.212	0.0001
Extraversion (EXTRA)	-0.235	0.0001	-0.179	0.002
Agreeableness (AGREE)	-0.254	0.0001	-0.335	0.0001
Conscientiousness (CONSC)	-0.261	0.0001	-0.189	0.001

The T-IL shows comparable scores for males and females, revealing significant correlations with social interaction anxiety, social phobia, well-being, and neuroticism. This underscores its relevance in understanding emotional and psychological states related to social isolation. Similarly, the Medical Student Well-Being Index (MSWBI) indicates similar perceptions of well-being among both genders, with significant correlations with social interaction anxiety, social phobia, loneliness, and neuroticism, highlighting their interconnected impact on medical students' emotional health, as given in Table 5.

**Table 5:** Correlation of social media addiction with loneliness and well-being T-ILS: Three-Item Loneliness Scale; MSWBI: Medical Student Well-Being Index Scale; NEURO: Neuroticism Spearman's Rho test is used for correlation between various factors. p < 0.05 is significant

Factor	Spearman’s Rho (T-ILS)	p-value (T-ILS)	Spearman’s Rho (MSWBI)	p-value (MSWBI)
Social interaction anxiety	0.498	0.0001	0.182	0.002
Social phobia	0.439	0.0001	0.232	0.0001
Well-being (MSWBI)	0.349	0.0001	-	-
Loneliness (T-ILS)	-	-	0.349	0.0001
Neuroticism (NEURO)	0.358	0.0001	0.214	0.0001

The regression analysis indicates that time spent on social media (time soc, coefficient: 4.312, p = 0.016) and agreeableness (AGREE, coefficient: 1.841, p = 0.040) are significant predictors of the dependent variable. Both predictors show no multicollinearity issues, with tolerance values at 0.993 and variance inflation factor (VIF) at 1.007. The intercept is 53.297 (p < 0.001), representing the baseline level of the dependent variable as given in Table 6.

**Table 6:** Multiple regression analysis of predictors of Social Media Addiction Scale-Student form VIF: variance inflation factor; SMA-SF: Social Media Addiction Scale-Student Form; time soc: time spent socializing multiple regression analysis; p < 0.05 is significant

Model	Unstandardized coefficients		Standardized coefficients	t	Sig.	95% confidence interval for B		Collinearity statistics		
	B	Std. error	Beta			Lower bound	Upper bound	Tolerance		VIF
Constant	53.235	7.188		7.406	0.000	39.087	67.383			
Time soc	4.392	1.769	0.144	2.483	0.014	0.910	7.873	0.993	1.007	
AGREE	1.840	0.890	0.120	2.068	0.040	0.089	3.590	0.993	1.007	
a. Dependent variable: SMA-SF										

## Discussion

In this study, participants predominantly belong to the early 20s age group, with an average age of 23.8 years and a standard deviation of 2.09 years. Both males (82.8%) and females (88.2%) were largely represented in the ≤23 age bracket, indicating a consistent age distribution across the cohort. Statistical analysis found no significant correlation between age group and gender (p = 0.1862), aligning with findings from Andreassen et al. [17], who observed no significant age-gender interaction in their study on social media addiction among young adults.

Regarding relationship status, a significant majority of participants, both males (83.6%) and females (77.6%), reported being single. Chi-square analysis (p = 0.4449) showed no significant association between relationship status and gender, echoing findings by Koc and Gulyagci [18] among Turkish college students. Their research indicated that relationship status did not influence susceptibility to social media addiction across genders.

Participants reported varying amounts of time spent on social media, with no statistically significant differences between males and females (p = 0.1295). This suggests similar usage patterns across genders, which aligns with Sha et al.'s [19] findings that gender does not strongly correlate with differences in social media usage time among young adults.

Analysis of the age at first phone acquisition showed a mean age of 15.92 years, with no significant gender differences (p = 0.1295). This reflects random variations rather than gender-based trends, as also noted by Twenge and Campbell [7] in their study on smartphone acquisition among youth. Finally, no significant association was found between gender and phone use before sleep (p = 0.4235), indicating consistent nighttime phone habits across genders, as found in research by Elhai et al. [20]. These studies collectively suggest that while individuals may vary in their digital habits, gender does not strongly dictate these behaviors related to social media use and smartphone habits.

This study investigated the relationship between social media addiction, social interaction anxiety (SIAS), and social phobia (SPS) among 292 participants. Significant positive correlations were observed between SMA-SF scores and both SIAS and SPS scores, indicating that higher social media addiction corresponded with increased levels of anxiety and phobia. Gender comparisons revealed no significant differences in SIAS and SPS scores between males and females, suggesting similar experiences across genders.

Further analysis showed that SIAS and SPS scores were associated with increased loneliness and neurotic tendencies. Conversely, these scores were negatively correlated with well-being, extraversion, agreeableness, and conscientiousness, indicating lower levels of these positive traits among individuals with higher social anxiety and phobia.

These findings are consistent with recent research. Huang's meta-analysis [21] and Vannucci et al. [22] demonstrated similar positive relationships between problematic social media usage and anxiety symptoms among young adults, supporting our study's observations. Gender comparisons align with Chan [23], indicating comparable levels of anxiety and phobia across genders related to social media use. Additionally, Dhir et al. [24] found similar correlations between social media addiction and personality traits, confirming our study's findings regarding neuroticism, loneliness, well-being, extraversion, agreeableness, and conscientiousness.

However, recent work by Orben et al. (2019) offers a contrasting perspective. Their large-scale analysis found that moderate to slightly high social media use had little to no significant impact on anxiety or phobia symptoms. This contradicts the strong positive relationships observed in our study, particularly concerning social media addiction and its association with anxiety and social phobia. Orben et al. suggest that the negative effects of social media on mental health might be overstated, with factors like excessive use or pre-existing conditions playing a larger role. This discrepancy may be explained by the focus of our study on addictive behaviors, which represent the end of social media usage, while Orben et al. analyze more moderate usage that may not necessarily lead to mental health issues [25].

In our study of 292 individuals, we identified a significant positive correlation between social media addiction, as measured by the SMA-SF, and loneliness assessed with the T-ILS, indicating that higher social media use is associated with increased feelings of loneliness. Gender comparisons revealed no significant differences in loneliness levels between males and females, suggesting a consistent experience across genders. Additionally, loneliness demonstrated significant positive associations with social interaction anxiety and social phobia; this suggests that individuals with higher levels of loneliness experience increased social anxiety and phobia. Conversely, loneliness was negatively correlated with well-being and neuroticism, indicating that higher levels of loneliness are linked to lower well-being and higher neurotic tendencies. Recent studies by Bányai et al. [26], Hawi and Samaha [27], and Kuss and Griffiths [28] have similarly identified associations between social media use and increased loneliness, highlighting the importance of comprehensive interventions to address these psychological challenges.

SMA-SF relates to overall well-being using the Medical Student Well-Being Index (MSWBI). We found a significant negative correlation between SMA-SF scores and MSWBI scores, indicating that higher social media addiction is linked to lower well-being. While gender differences in well-being perception among medical students were noted, they did not reach statistical significance.

Recent studies have reinforced these findings. Błachnio et al. [29] identified a significant association between Facebook addiction and diminished self-esteem and life satisfaction, highlighting the negative impacts of excessive social media use on personal well-being. De Choudhury and Kiciman [30] investigated social support language on social media, noting that while beneficial, excessive use often correlates with increased anxiety and reduced well-being. Similarly, Dhir et al. [24] explored compulsive social media use's impact on psychological health, revealing significant negative effects on well-being. These studies underscore the importance of addressing social media addiction to promote better mental health outcomes.

The study investigated social media addiction using the SMA-SF and its correlation with personality traits measured by the BFI. It found no gender differences in addiction levels (p = 0.801), consistent with findings by Al-Menayes [31] among Saudi university students. Agreeableness showed a slight positive association with addiction, while conscientiousness had a negative correlation, as noted by Valkenburg and Peter [32]. Less extroverted individuals were more prone to addiction, similar to patterns found by Błachnio et al. [29], while higher neuroticism correlated positively with addiction, akin to research by Dhir et al. [24] and Kuss and Lopez-Fernandez [33]. Openness to experience did not significantly correlate with addiction, in line with Gosling et al.'s study [34]. These findings underscore the need for personalized interventions based on individual personality profiles to manage social media addiction effectively.

Additionally, as Schmitt et al. [35] pointed out, males tend to score higher in extraversion and assertiveness compared to females, which could influence their engagement with social media and addictive behaviors. However, in this study, these personality differences did not result in significant gender variations in addiction levels. These findings underscore the need for personalized interventions based on individual personality profiles to manage social media addiction effectively.

The study investigated predictors of social media addiction using the SMA-SF, identifying time soc and agreeableness (AGREE) as significant factors. Increased time soc predicted higher SMA-SF scores, indicating a link between socializing and addiction. Similarly, higher AGREE scores correlated with elevated addiction levels. The regression model, validated by ANOVA, explained variance in SMA-SF scores significantly, with time soc and AGREE positively influencing scores. Recent studies by Király et al. [36] highlighted social factors in addiction, while Burke et al. [37] emphasized personality traits as predictors of addiction, emphasizing its complex nature.

Strength

The study participants were from a center that had students from across the country. Thus, the findings are significant.

Limitation

The study was conducted at a single center, and the use of Google Forms could have created question bias.

Implication

The high prevalence of social media addiction among medical students highlights the urgent need for targeted interventions that could significantly improve their overall well-being and mental health. This underscores the importance of further research and the development of effective strategies within academic environments.

## Conclusions

This study reveals a significant prevalence of social media addiction among medical students, with 76.7% (n = 224) showing signs of addiction, slightly higher among females (n = 132, 78%) compared to males (n = 92, 72%). This addiction correlates with adverse psychological traits such as heightened social interaction anxiety, social phobia, and loneliness, alongside personality traits like neuroticism. It is also associated with lower levels of extraversion, agreeableness, and conscientiousness. Both genders showed similar susceptibility to addiction, influenced by factors such as socializing time and agreeableness.
